# Crystal structure of *N*,*N*′-bis­(diiso­propyl­phosphan­yl)-4-methyl­pyridine-2,6-di­amine

**DOI:** 10.1107/S1600536814010976

**Published:** 2014-08-01

**Authors:** Berthold Stöger, Matthias Weil, Bernhard Bichler, Wolfgang Eder, Karl Kirchner

**Affiliations:** a Institute for Chemical Technologies and Analytics, Division of Structural Chemistry, Vienna University of Technology, Getreidemarkt 9/164-SC, A-1060 Vienna, Austria; b Institute of Applied Synthetic Chemistry, Vienna University of Technology, Getreidemarkt 9/163, A-1060 Vienna, Austria

**Keywords:** crystal structure, PNP pincer ligand, methylpyridine-2,6-diamine

## Abstract

In the mol­ecule of the title compound, C_18_H_35_N_3_P_2_, the methyl­pyridine-2,6-di­amine moiety is almost planar, with a maximum deviation of 0.0129 (9) Å for one of the amine N atoms. Whereas one of the P atoms is co-planar with this mean plane [deviation = 0.0158 (10) Å], the other P atom is considerably displaced out of the mean plane by 0.5882 (10) Å. In the crystal, no directional intermolecular interactions beyond van der Waals contacts could be identified.

## Related literature   

The title compound belongs to the family of PNP pincer ligands that are capable of forming complexes with various transition metals, leading to inter­esting properties and applications, see: Benito-Garagorri & Kirchner (2008 ▶); Langer *et al.* (2011 ▶); Bichler *et al.* (2013 ▶). For general aspects of pincer ligands and derived complexes, see: Morales-Morales & Jensen (2007 ▶).
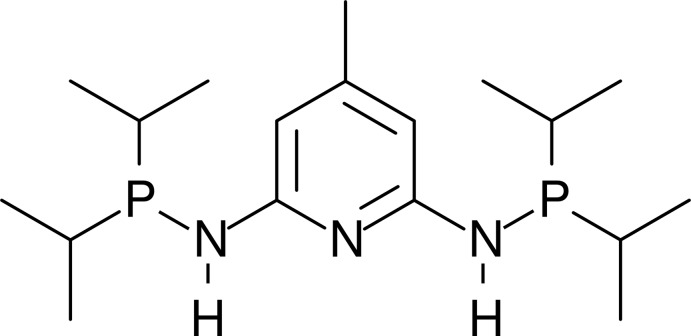


## Experimental   

### Crystal data  

C_18_H_35_N_3_P_2_
*M* *_r_* = 355.4
Orthorhombic, 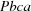

*a* = 14.3394 (12) Å
*b* = 10.0089 (16) Å
*c* = 29.562 (3) Å
*V* = 4242.9 (9) Å^3^
*Z* = 8
Mo *K*α radiation
μ = 0.21 mm^−1^
*T* = 100 K
0.70 × 0.28 × 0.04 mm

### Data collection  

Bruker APEXII CCD diffractometer
Absorption correction: multi-scan (*SADABS*; Bruker, 2013 ▶) *T*_min_ = 0.93, *T*_max_ = 0.99
149837 measured reflections
6241 independent reflections
4614 reflections with *I* > 3σ(*I*)
*R*_int_ = 0.066

### Refinement  

*R*[*F* ^2^ > 2σ(*F* ^2^)] = 0.031
*wR*(*F* ^2^) = 0.046
*S* = 1.39
6241 reflections
216 parameters
2 restraints
H atoms treated by a mixture of independent and constrained refinement
Δρ_max_ = 0.29 e Å^−3^
Δρ_min_ = −0.21 e Å^−3^

### 

Data collection: *APEX2* (Bruker, 2013 ▶); cell refinement: *SAINT-Plus* (Bruker, 2013 ▶); data reduction: *SAINT-Plus*; program(s) used to solve structure: *SUPERFLIP* (Palatinus & Chapuis, 2007 ▶); program(s) used to refine structure: *JANA2006* (Petříček, *et al.*, 2014 ▶); molecular graphics: *Mercury* (Macrae *et al.*, 2008 ▶); software used to prepare material for publication: *publCIF* (Westrip, 2010 ▶).

## Supplementary Material

Crystal structure: contains datablock(s) global, I. DOI: 10.1107/S1600536814010976/su0004sup1.cif

Structure factors: contains datablock(s) I. DOI: 10.1107/S1600536814010976/su0004Isup2.hkl

Supporting information file. DOI: 10.1107/S1600536814010976/su0004Isup3.cml

**Fig. 1.**
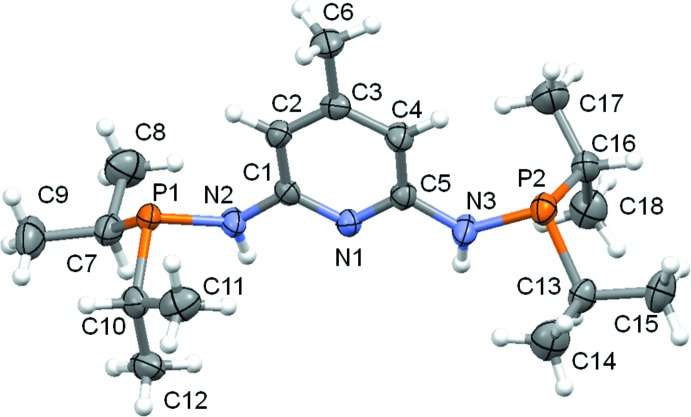
The molecular structure of the title compound, with atom labelling. Displacement ellipsoids are drawn at the 80% probability level.

. DOI: 10.1107/S1600536814010976/su0004fig1.tif
The mol­ecular structure of the title compound, with atom labelling. Displacement ellipsoids are drawn at the 80% probability level.

CCDC reference: 1004282

Additional supporting information:  crystallographic information; 3D view; checkCIF report

## supplementary crystallographic information

### S1. Experimental

4-Methylpyridine-2,6-diamine (500 mg, 4 mmol, 1 eq) was diluted in toluene and triethylamine (1.2 ml, 8.6 mmol, 2.1 eq) was added. The mixture was cooled to 273 K using an ice bath and chlorodiisopropylphosphine (1.3 ml, 8.2 mmol, 2.05 eq) was added drop wise using a syringe. The cooling bath was removed, and after reaching room temperature, the reaction mixture was stirred at 353 K for another 12 h. The precipitate was filtered off and the solvent was evaporated. Yield: 69% off-white powder. For further purification, the compound was recrystallized using a toluene/hexane 1:1 (*v/v*) mixture. Spectroscopic data for the title compound are available in the archived CIF.

### S2. Refinement

The H atoms of the amine groups were located in difference Fourier maps and the N—H distances restrained to 0.87 Å. The remaining H atoms were placed geometrically and refined as riding on the parent C atoms with *U*_iso_(H) =1.2*U*_eq_(C).

### Crystal data

**Table:**

C_18_H_35_N_3_P_2_	*F*(000) = 1552
*M**_r_* = 355.4	*D*_x_ = 1.113 Mg m^−^^3^
Orthorhombic, *P**b**c**a*	Mo *K*α radiation, λ = 0.71073 Å
Hall symbol: -P 2ac 2ab	Cell parameters from 9974 reflections
*a* = 14.3394 (12) Å	θ = 2.5–30°
*b* = 10.0089 (16) Å	µ = 0.21 mm^−^^1^
*c* = 29.562 (3) Å	*T* = 100 K
*V* = 4242.9 (9) Å^3^	Plate, translucent colourless
*Z* = 8	0.70 × 0.28 × 0.04 mm

### Data collection

**Table:**

Bruker APEXII CCD diffractometer	6241 independent reflections
Radiation source: X-ray tube	4614 reflections with *I* > 3σ(*I*)
Graphite monochromator	*R*_int_ = 0.066
ω and φ scans	θ_max_ = 30.1°, θ_min_ = 1.4°
Absorption correction: multi-scan (*SADABS*; Bruker, 2013)	*h* = −20→20
*T*_min_ = 0.93, *T*_max_ = 0.99	*k* = −14→14
149837 measured reflections	*l* = −41→41

### Refinement

**Table:**

Refinement on *F*	Primary atom site location: iterative
*R*[*F*^2^ > 2σ(*F*^2^)] = 0.031	Secondary atom site location: none
*wR*(*F*^2^) = 0.046	Hydrogen site location: geom,difmap
*S* = 1.39	H atoms treated by a mixture of independent and constrained refinement
6241 reflections	Weighting scheme based on measured s.u.'s *w* = 1/(σ^2^(*F*) + 0.0004*F*^2^)
216 parameters	(Δ/σ)_max_ = 0.014
2 restraints	Δρ_max_ = 0.29 e Å^−^^3^
132 constraints	Δρ_min_ = −0.21 e Å^−^^3^

### Special details

**Table:**

Experimental. Spectroscopic data for the title compound:^1^H-NMR (400 MHz, CDCl_3_) 6.34 (s, 2H), 4.88 (bs, 2H), 2.18 (s, 3H), 1.87–1.60 (m, 4H), 1.15–0.92 (m, 24H). ^31^P NMR (162 MHz, CDCl_3_) 55.65. ^13^C-NMR (63 MHz, CDCl_3_) 156.59 (d, *J* = 21.9 Hz), 153.94 (*s*), 99.14 (d, *J* = 19.7 Hz), 26.27 (d, *J* = 11.4 Hz), 21.80 (d, *J* = 15.3 Hz), 18.46 (d, *J* = 19.7 Hz), 17.18 (d, *J* = 7.9 Hz).

### Fractional atomic coordinates and isotropic or equivalent isotropic displacement parameters (Å^2^)

**Table:**

	*x*	*y*	*z*	*U*_iso_*/*U*_eq_	
P1	0.781952 (19)	0.61761 (3)	0.277629 (10)	0.01628 (8)	
P2	0.62798 (2)	0.33177 (3)	0.045775 (10)	0.01837 (8)	
N1	0.70180 (6)	0.42925 (9)	0.16952 (3)	0.0149 (2)	
N2	0.73728 (7)	0.50053 (10)	0.24153 (3)	0.0176 (3)	
N3	0.66959 (7)	0.33964 (10)	0.10001 (3)	0.0200 (3)	
C1	0.69814 (7)	0.52954 (11)	0.19953 (4)	0.0146 (3)	
C2	0.65663 (7)	0.65213 (11)	0.19012 (4)	0.0166 (3)	
C3	0.61705 (7)	0.67148 (11)	0.14781 (4)	0.0164 (3)	
C4	0.62017 (8)	0.56884 (11)	0.11637 (4)	0.0180 (3)	
C5	0.66312 (7)	0.44882 (11)	0.12890 (4)	0.0158 (3)	
C6	0.57228 (8)	0.80360 (11)	0.13652 (4)	0.0220 (3)	
C7	0.76791 (8)	0.52642 (12)	0.33135 (4)	0.0211 (3)	
C8	0.66449 (9)	0.50783 (15)	0.34168 (5)	0.0326 (4)	
C9	0.81669 (11)	0.60387 (14)	0.36929 (4)	0.0321 (4)	
C10	0.90932 (8)	0.60456 (12)	0.26658 (4)	0.0207 (3)	
C11	0.92671 (9)	0.64908 (13)	0.21773 (4)	0.0280 (4)	
C12	0.95354 (8)	0.46787 (12)	0.27535 (4)	0.0251 (3)	
C13	0.71074 (8)	0.20717 (12)	0.02331 (4)	0.0196 (3)	
C14	0.80702 (9)	0.27110 (14)	0.01819 (4)	0.0304 (4)	
C15	0.67652 (9)	0.15302 (14)	−0.02210 (4)	0.0288 (4)	
C16	0.51998 (8)	0.23173 (12)	0.05297 (4)	0.0218 (3)	
C17	0.44882 (9)	0.31763 (15)	0.07830 (5)	0.0322 (4)	
C18	0.53214 (9)	0.09667 (13)	0.07581 (4)	0.0285 (4)	
H1c2	0.655417	0.721798	0.212436	0.0199*	
H1c4	0.593587	0.579767	0.086795	0.0216*	
H1c6	0.531526	0.829737	0.160715	0.0264*	
H2c6	0.619791	0.870155	0.132575	0.0264*	
H3c6	0.536997	0.795035	0.109071	0.0264*	
H1c7	0.795933	0.439591	0.328992	0.0253*	
H1c8	0.634885	0.463114	0.316796	0.0392*	
H2c8	0.657585	0.4551	0.368598	0.0392*	
H3c8	0.635919	0.593558	0.346145	0.0392*	
H1c9	0.88155	0.614221	0.362035	0.0386*	
H2c9	0.788416	0.690318	0.372402	0.0386*	
H3c9	0.810676	0.555582	0.397193	0.0386*	
H1c10	0.939505	0.661523	0.288221	0.0248*	
H1c11	0.897622	0.734181	0.212703	0.0336*	
H2c11	0.992597	0.656607	0.212556	0.0336*	
H3c11	0.900823	0.584359	0.197319	0.0336*	
H1c12	0.950231	0.447657	0.307053	0.0302*	
H2c12	0.920525	0.400794	0.258548	0.0302*	
H3c12	1.017612	0.469408	0.265948	0.0302*	
H1c13	0.714996	0.133665	0.044067	0.0235*	
H1c14	0.823637	0.315444	0.045839	0.0365*	
H2c14	0.852243	0.203124	0.011583	0.0365*	
H3c14	0.805635	0.334884	−0.006051	0.0365*	
H1c15	0.620148	0.103025	−0.0176	0.0345*	
H2c15	0.664577	0.226133	−0.042306	0.0345*	
H3c15	0.723356	0.095883	−0.034922	0.0345*	
H1c16	0.498808	0.208867	0.023147	0.0262*	
H1c17	0.440136	0.400466	0.062477	0.0386*	
H2c17	0.390461	0.270903	0.080008	0.0386*	
H3c17	0.471218	0.335423	0.108302	0.0386*	
H1c18	0.569613	0.039846	0.056995	0.0343*	
H2c18	0.562348	0.108617	0.104509	0.0343*	
H3c18	0.472136	0.056297	0.080352	0.0343*	
H1n3	0.6939 (10)	0.2689 (9)	0.1124 (5)	0.042 (4)*	
H1n2	0.7548 (9)	0.4176 (4)	0.2438 (5)	0.027 (4)*	

### Atomic displacement parameters (Å^2^)

**Table:**

	*U*^11^	*U*^22^	*U*^33^	*U*^12^	*U*^13^	*U*^23^
P1	0.01745 (13)	0.01506 (14)	0.01634 (13)	0.00115 (10)	−0.00312 (10)	−0.00393 (11)
P2	0.02390 (15)	0.01747 (15)	0.01375 (13)	0.00096 (11)	−0.00236 (10)	−0.00068 (11)
N1	0.0140 (4)	0.0141 (4)	0.0165 (4)	−0.0004 (3)	−0.0013 (3)	−0.0016 (3)
N2	0.0208 (5)	0.0137 (4)	0.0182 (4)	0.0012 (4)	−0.0056 (4)	−0.0025 (4)
N3	0.0287 (5)	0.0153 (5)	0.0160 (4)	0.0045 (4)	−0.0058 (4)	−0.0028 (4)
C1	0.0112 (4)	0.0159 (5)	0.0167 (5)	−0.0022 (4)	−0.0006 (4)	−0.0010 (4)
C2	0.0156 (5)	0.0141 (5)	0.0199 (5)	0.0001 (4)	−0.0007 (4)	−0.0034 (4)
C3	0.0139 (5)	0.0135 (5)	0.0217 (5)	−0.0003 (4)	0.0000 (4)	0.0010 (4)
C4	0.0207 (5)	0.0163 (5)	0.0170 (5)	0.0016 (4)	−0.0031 (4)	0.0002 (4)
C5	0.0156 (5)	0.0155 (5)	0.0162 (5)	−0.0014 (4)	0.0003 (4)	−0.0020 (4)
C6	0.0239 (6)	0.0168 (5)	0.0253 (6)	0.0045 (4)	−0.0025 (4)	0.0002 (5)
C7	0.0249 (6)	0.0211 (6)	0.0171 (5)	0.0032 (4)	−0.0013 (4)	−0.0022 (4)
C8	0.0296 (6)	0.0423 (8)	0.0260 (6)	0.0022 (6)	0.0067 (5)	0.0028 (6)
C9	0.0461 (8)	0.0318 (7)	0.0185 (6)	0.0040 (6)	−0.0077 (5)	−0.0039 (5)
C10	0.0177 (5)	0.0183 (6)	0.0260 (6)	−0.0018 (4)	−0.0031 (4)	−0.0044 (5)
C11	0.0260 (6)	0.0249 (6)	0.0332 (7)	−0.0011 (5)	0.0065 (5)	0.0012 (5)
C12	0.0182 (5)	0.0240 (6)	0.0332 (7)	0.0018 (4)	−0.0041 (5)	−0.0020 (5)
C13	0.0227 (5)	0.0198 (5)	0.0161 (5)	−0.0014 (4)	0.0030 (4)	−0.0015 (4)
C14	0.0269 (6)	0.0341 (8)	0.0301 (7)	−0.0059 (5)	0.0072 (5)	−0.0027 (6)
C15	0.0374 (7)	0.0300 (7)	0.0189 (6)	−0.0014 (6)	0.0031 (5)	−0.0060 (5)
C16	0.0192 (5)	0.0272 (6)	0.0191 (5)	0.0018 (5)	−0.0018 (4)	−0.0049 (5)
C17	0.0242 (6)	0.0400 (8)	0.0323 (7)	0.0051 (6)	0.0006 (5)	−0.0097 (6)
C18	0.0271 (6)	0.0282 (7)	0.0303 (7)	−0.0062 (5)	0.0044 (5)	−0.0002 (5)

### Geometric parameters (Å, º)

**Table:**

P1—N2	1.7094 (10)	C9—H2c9	0.96
P1—C7	1.8426 (12)	C9—H3c9	0.96
P1—C10	1.8599 (12)	C10—C11	1.5319 (18)
P2—N3	1.7125 (11)	C10—C12	1.5300 (17)
P2—C13	1.8452 (12)	C10—H1c10	0.96
P2—C16	1.8564 (12)	C11—H1c11	0.96
N1—C1	1.3408 (14)	C11—H2c11	0.96
N1—C5	1.3372 (14)	C11—H3c11	0.96
N2—C1	1.3932 (14)	C12—H1c12	0.96
N2—H1n2	0.870 (6)	C12—H2c12	0.96
N3—C5	1.3901 (15)	C12—H3c12	0.96
N3—H1n3	0.870 (11)	C13—C14	1.5292 (18)
C1—C2	1.3918 (16)	C13—C15	1.5286 (17)
C2—C3	1.3872 (16)	C13—H1c13	0.96
C2—H1c2	0.96	C14—H1c14	0.96
C3—C4	1.3861 (16)	C14—H2c14	0.96
C3—C6	1.5074 (16)	C14—H3c14	0.96
C4—C5	1.3998 (16)	C15—H1c15	0.96
C4—H1c4	0.96	C15—H2c15	0.96
C6—H1c6	0.96	C15—H3c15	0.96
C6—H2c6	0.96	C16—C17	1.5301 (18)
C6—H3c6	0.96	C16—C18	1.5212 (18)
C7—C8	1.5256 (17)	C16—H1c16	0.96
C7—C9	1.5324 (18)	C17—H1c17	0.96
C7—H1c7	0.96	C17—H2c17	0.96
C8—H1c8	0.96	C17—H3c17	0.96
C8—H2c8	0.96	C18—H1c18	0.96
C8—H3c8	0.96	C18—H2c18	0.96
C9—H1c9	0.96	C18—H3c18	0.96
			
N2—P1—C7	99.06 (5)	P1—C10—C12	116.11 (8)
N2—P1—C10	102.14 (5)	P1—C10—H1c10	106.5
C7—P1—C10	102.93 (5)	C11—C10—C12	110.63 (10)
N3—P2—C13	98.28 (5)	C11—C10—H1c10	112.45
N3—P2—C16	102.02 (5)	C12—C10—H1c10	103.38
C13—P2—C16	102.31 (5)	C10—C11—H1c11	109.47
C1—N1—C5	117.93 (9)	C10—C11—H2c11	109.47
P1—N2—C1	124.36 (8)	C10—C11—H3c11	109.47
P1—N2—H1n2	119.8 (9)	H1c11—C11—H2c11	109.47
C1—N2—H1n2	112.7 (9)	H1c11—C11—H3c11	109.47
P2—N3—C5	126.02 (8)	H2c11—C11—H3c11	109.47
P2—N3—H1n3	119.7 (9)	C10—C12—H1c12	109.47
C5—N3—H1n3	114.1 (9)	C10—C12—H2c12	109.47
N1—C1—N2	114.71 (9)	C10—C12—H3c12	109.47
N1—C1—C2	122.99 (10)	H1c12—C12—H2c12	109.47
N2—C1—C2	122.29 (10)	H1c12—C12—H3c12	109.47
C1—C2—C3	118.57 (10)	H2c12—C12—H3c12	109.47
C1—C2—H1c2	120.71	P2—C13—C14	109.48 (9)
C3—C2—H1c2	120.71	P2—C13—C15	110.44 (8)
C2—C3—C4	119.21 (10)	P2—C13—H1c13	109.19
C2—C3—C6	119.76 (10)	C14—C13—C15	110.57 (10)
C4—C3—C6	121.02 (10)	C14—C13—H1c13	109.06
C3—C4—C5	118.21 (10)	C15—C13—H1c13	108.06
C3—C4—H1c4	120.89	C13—C14—H1c14	109.47
C5—C4—H1c4	120.89	C13—C14—H2c14	109.47
N1—C5—N3	114.13 (9)	C13—C14—H3c14	109.47
N1—C5—C4	123.08 (10)	H1c14—C14—H2c14	109.47
N3—C5—C4	122.78 (10)	H1c14—C14—H3c14	109.47
C3—C6—H1c6	109.47	H2c14—C14—H3c14	109.47
C3—C6—H2c6	109.47	C13—C15—H1c15	109.47
C3—C6—H3c6	109.47	C13—C15—H2c15	109.47
H1c6—C6—H2c6	109.47	C13—C15—H3c15	109.47
H1c6—C6—H3c6	109.47	H1c15—C15—H2c15	109.47
H2c6—C6—H3c6	109.47	H1c15—C15—H3c15	109.47
P1—C7—C8	109.83 (8)	H2c15—C15—H3c15	109.47
P1—C7—C9	109.29 (8)	P2—C16—C17	108.02 (9)
P1—C7—H1c7	109.89	P2—C16—C18	115.76 (8)
C8—C7—C9	111.03 (10)	P2—C16—H1c16	106.69
C8—C7—H1c7	108.11	C17—C16—C18	111.01 (10)
C9—C7—H1c7	108.66	C17—C16—H1c16	111.87
C7—C8—H1c8	109.47	C18—C16—H1c16	103.42
C7—C8—H2c8	109.47	C16—C17—H1c17	109.47
C7—C8—H3c8	109.47	C16—C17—H2c17	109.47
H1c8—C8—H2c8	109.47	C16—C17—H3c17	109.47
H1c8—C8—H3c8	109.47	H1c17—C17—H2c17	109.47
H2c8—C8—H3c8	109.47	H1c17—C17—H3c17	109.47
C7—C9—H1c9	109.47	H2c17—C17—H3c17	109.47
C7—C9—H2c9	109.47	C16—C18—H1c18	109.47
C7—C9—H3c9	109.47	C16—C18—H2c18	109.47
H1c9—C9—H2c9	109.47	C16—C18—H3c18	109.47
H1c9—C9—H3c9	109.47	H1c18—C18—H2c18	109.47
H2c9—C9—H3c9	109.47	H1c18—C18—H3c18	109.47
P1—C10—C11	107.76 (8)	H2c18—C18—H3c18	109.47
