# Supplementary figures and images for: Crystal structure of N,N′-bis­(diiso­propyl­phosphan­yl)-4-methyl­pyridine-2,6-di­amine
Source: Acta Crystallogr Sect E Struct Rep Online. 2014 Aug 1;70(Pt 9):o889–90. doi: 10.1107/S1600536814010976 (PMC4186153; doi:10.1107/S1600536814010976)

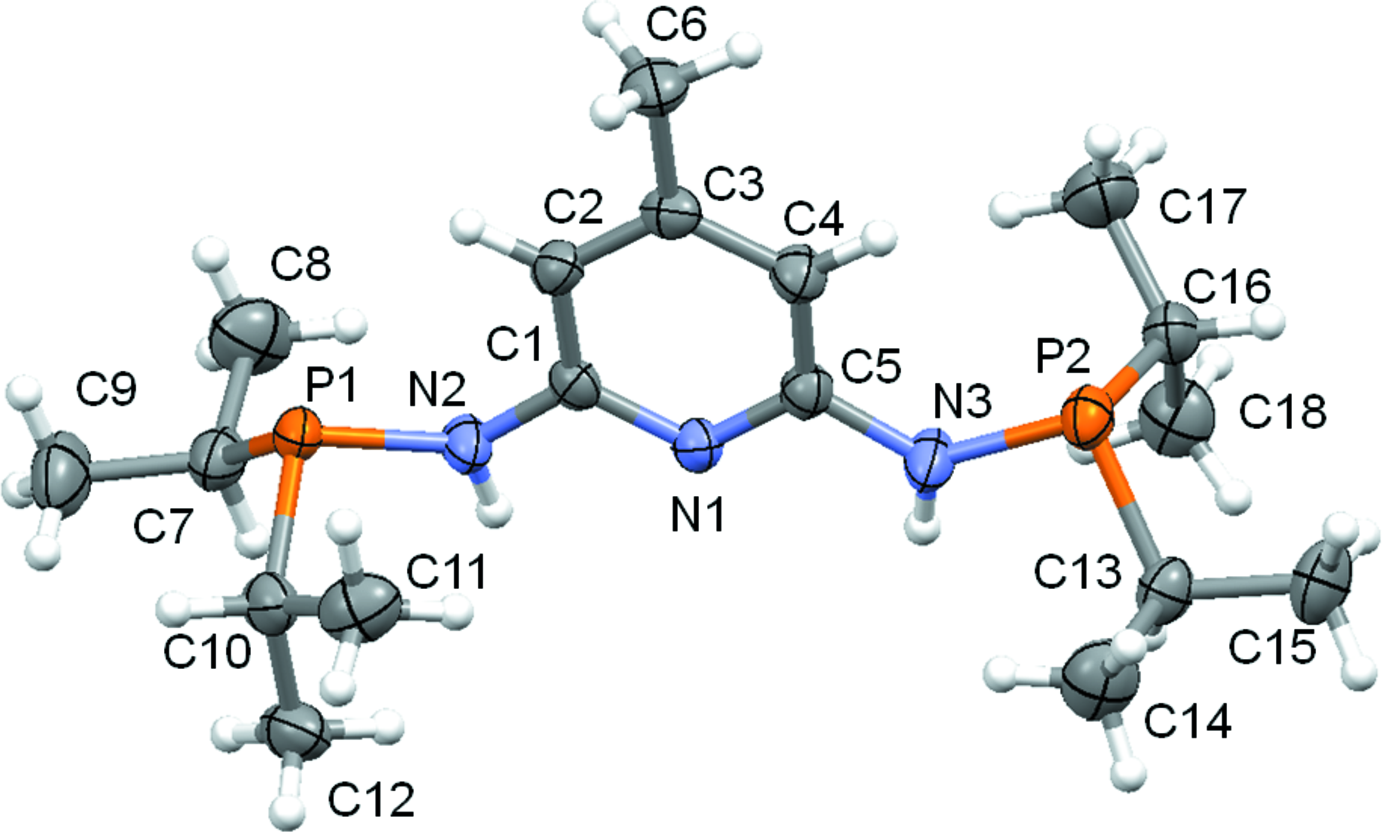

Supplement: Supplementary file 4 [file e-70-0o889-fig1.tif]
